# Drug Insurance and Psoriasis Severity: A Retrospective Cohort Study

**DOI:** 10.36469/001c.127820

**Published:** 2025-02-07

**Authors:** Laurence Mainville, Hélène Veillette, Paul R. Fortin

**Affiliations:** 1 Division of Dermatology Department of Medicine, CHU de Québec, Université Laval, Québec, Canada; 2 Medicine Department of Medicine, CHU de Québec-Université Laval; 3 Division of Rheumatology Department of Medicine, CHU de Québec, Université Laval, Québec, Canada; 4 Centre de recherche du CHU de Québec – Université Laval

**Keywords:** dermatology, psoriasis, advanced therapy, drug coverage, clinical epidemiology

## Abstract

**Background:** Prescription drug insurance in Canada is constituted of a patchwork of public and private insurance plans. The type of drug insurance may have a negative impact on access to treatment for patients covered by public plans compared with private plans. **Objectives:** In patients with psoriasis treated with advanced therapy in public vs private drug insurance groups, we compared: (1) psoriasis severity scores when an advanced therapy was prescribed, (2) psoriasis severity scores at follow-up, (3) treatment response, and (4) delay between prescription and first dose of advanced therapy. **Methods:** This unicentric, retrospective cohort study included patients suffering from psoriasis treated by advanced therapy, dermatologist-prescribed between September 2015 and August 2019, in a tertiary academic care center in Québec City, Canada. Data were collected from medical records. **Results:** Patients treated with an advanced therapy for psoriasis covered under the provincial public drug insurance plan (n = 78) and under a private drug plan (n = 93) did not differ regarding the studied outcomes. Patients’ characteristics differed between groups. Patients in the public group were older (*P* < .0001), more socioeconomically deprived (*P* < .05), and more likely to benefit from compassion from the industry to access a prescribed medication free of charge (*P* < .0001) compared with patients from the privately insured group. **Discussion:** The high prevalence of compassionate programs from the industry in the public insurance group (42% vs 14%), and the high prevalence of psoriasis on difficult-to-treat areas (face, genitalia, and/or palmoplantar areas) in our cohort (85.4%) may mask differences in access to advanced therapy between the two groups. **Conclusions:** Prescribers of advanced therapy can be reassured, as we found no inequality in access or care based on patients’ drug insurance coverage.

## INTRODUCTION

Psoriasis is a chronic inflammatory skin disease with an estimated prevalence of 1.7% in Canada.^1^ Access to advanced therapy is limited by high cost and complex cumulative criteria.^2–5^ In Québec, the annual cost of advanced therapy per individual in 2020 ranged from CAD$19 999 to $37 465 for year 1 of treatment.^6^ Canadians have public access to health care. Prescription drug insurance is a separate and complex regimen administered by both public and private insurers. Insurers in Québec and Canada require fulfillment of specific cumulative criteria before they make the decision to cover the costs of an advanced therapy for psoriasis: Psoriasis Area and Severity Index (PASI) ≥ 12, or the presence of large plaques on difficult-to-treat areas; no significant improvement after at least 30 sessions of phototherapy for 3 months or after at least 12 sessions for 1 month; failure of treatment with 2 conventional systemic treatments for after at least 3 months. Since this study was carried out, minor changes to reimbursement criteria have been made (eg, minimum required PASI score lowered from 15 to 12, without Dermatology Life Quality Index [DLQI] requirement), and 2 other advanced therapies (bimekizumab, deucravacitinib) became available.

German authors have advocated for more flexible regulations that would promote patient-centered, cost-effective care.^7^ Literature on access to advanced therapy using type of drug insurance as a predictor variable is mainly composed of observational data, with inconstant adjustment for major confounding covariates like socioeconomic status and compassionate care programs from the industry.^8–11^ In a Canadian study, Rumman et al observed that type of drug insurance was an independent predictor variable of access to biologic agents in inflammatory bowel disease (n = 268).^10^ In another study of 693 patients with rheumatoid arthritis conducted in the United States, Medicaid patients were less likely to receive biotherapy (adjusted odds ratio = 0.09; *P<* .001) than the private group. Results were adjusted for age, sex, and race, but not for socioeconomic status.^12^ Increased disease severity and longer time to start advanced therapy after its initial prescription were observed in various autoimmune and inflammatory conditions (lupus nephritis, rheumatoid arthritis, inflammatory bowel disease, psoriasis), suggesting a form of inequality in treatment access.^8,10,12,13^

We conducted a study to assess the access to advanced therapies (including biologic agents and small molecules) prescribed for psoriasis using the type of drug insurance as a predictor variable.

## METHODS

### Study Design

We conducted a unicentric retrospective cohort study of patients with psoriasis treated with a dermatologist-prescribed advanced therapy. The study was held in one of the academic hospitals of the CHU de Québec-Université Laval, and before biosimilar drugs were available in Canada. Patients were divided in two groups: public prescription drug insurance plan (public), and private prescription drug insurance plan (private).

### Study Population and Data Source

Given the limited risk for study participants, broad inclusion criteria were established. We included all patients with a confirmed clinical diagnosis of psoriasis started on an advanced therapy prescribed by a dermatologist at a single hospital between September 2015 and August 2019. Patients were excluded if treatment indication did not include psoriasis. Patients with missing data and prior advanced therapy outside the study period were also considered for inclusion. To facilitate the study of psoriasis in our center, a retrospective study design was favored to work from a convenience sample issued of the practice of dermatologists. The rationale for a cohort study was to allow comparison of severity scores over a follow-up period.

### Study Variables

The predictor variable was the type of prescription drug insurance (public vs private) at the time of advanced-therapy prescription (index date). Dependent variables included PASI, a validated physician-reported outcome used to score the severity and extent of psoriasis (0-72)^1,14^; the DLQI, a validated patient quality-of-life self-administered questionnaire specific to cutaneous problems (0-30)^1,15,16^; Investigator’s Global Assessment (IGA), a physician-administered validated tool used to assess psoriasis severity from 0 to 4.^1,17^ The PASI50, PASI75, and PASI90 indicate a 50%, 75%, and 90% decrease in PASI score between 2 time points, respectively.^1^ Finally, the delay to first dose of advanced therapy is a dependent variable representing the time (days) between index date and initiation of treatment with an advanced therapy.^10^

### Covariates

The variables capturing demographic and clinical characteristics at baseline and potential factors of influence on psoriasis were identified in the literature.^10,18^ Captured subtypes of psoriasis (eg, plaque, pustular, inverse, guttate psoriasis) were based on clinical assessment made by dermatologists. Compassion from the industry is a dichotomous variable captured from the “psoriasis summary sheet” within medical records (a hospital-based formulary updated by nursing staff).

The 2011 Deprivation Index Assignment Program is a SAS program, based on Canadian Census data and the National Household Survey.^19,20^ The index is subdivided into material and social components. Each index is subdivided into material (average income, proportion of individuals without a high school diploma, and proportion of employed individuals) and social components (proportion of individuals living alone, proportion of lone parent families, and proportion of separated, divorced, or widowed individuals). It assigns a deprivation index to any SAS file that includes a field with postal codes (6 digits) in Canada. We categorized the Deprivation Index quintiles to simplify our analyses of deprived (3rd, 4th, 5th quintiles = 0), vs socioeconomically privileged individuals (first, second quintiles = 1).

### Data Collection

One of the investigators (L.M.) used a standardized, unvalidated, anonymized collection grid to collect all data unblinded. In patients who received multiple advanced therapies, we used data regarding the oldest prescription during the study period.

### Statistical Analyses

**Sample size calculation:** Based on previous literature, we calculated a minimum sample size of 102 patients to provide 80% power and to detect a clinical difference of 5 points in PASI pre-treatment, with a 2-sided 5% level of significance.^21,22^ We used a standard deviation of 9 for PASI pre-treatment.^21,22^

**Statistical approach**: Means, standard deviations, medians, and ranges were used to describe continuous variables. Proportions were used to describe categorized variables. In univariate analyses, we used Student’s *t* test to compare psoriasis severity scores, treatment response, and delay between groups (n_1_ ≥ 30 and n_2_ ≥ 30), using a 5% level of significance. Pearson χ^2^ test was used to compare achievement of PASI50, PASI75, and PASI90 between groups (n ≥ 5) with a 5% level of significance. The variable delay to first dose of advanced therapy was log-transformed to control for abnormality of the distribution and extreme variables. The *t*-test statistics were performed on log-transformed data. Multivariate linear regression models were used to consider potential confounding factors using the ordinary least squares estimation method, with a 5% level of significance. Four explicative multivariate linear regression models were constructed using the following outcome variables: (1) PASI pre-treatment, (2) DLQI pre-treatment, (3) treatment response, and (4) log[delay]. All models used type of drug insurance as a predictor variable of interest (*X_i_*). Thirteen covariates were included in full models: age at prescription, sex, deprivation index (material component), deprivation index (social component), psoriasis subtype, psoriasis on difficult-to-treat areas, comorbidities, smoking status, biologic agent, previous exposure to biotherapy, compassion, and time between index date and renewal. Multivariate linear regression models were favored considering the linear relationship between the dichotomous variable type of drug insurance (*X_i_*) and the mean of *Y*. All linear regression model assumptions were verified and fulfilled. All statistical analyses were performed by L.M. using SAS^®^ Studio 3.8.

### Ethics

The project was approved by the CHU de Québec-Université Laval’s Ethics Committee in January 2020 (2020-5036), and reconducted until June 2025.

## RESULTS

### Patient Characteristics

Data were retrieved from the practice of 6 dermatologists from a tertiary care center in Québec City. We hand searched 314 medical records of patients on advanced therapy between September 2015 and June 2020. We included 171 individuals meeting our eligibility criteria. Patients’ demographic and clinical characteristics are outlined in **Table 1**. The public and private groups included 78 (46%) and 93 (54%) patients, respectively. The mean (SD) age at time of advanced therapy prescription was 55 (17) years in the public group, and 45 (13) years in the private group (*P <* .0001). The mean (SD) age at first psoriasis diagnosis was 34 (17) years and 30 (15) years in the public and private groups, respectively (*P =* .02). Most individuals presented with psoriasis on difficult-to-treat areas ([146] 85%). More than 1 comorbidity was noted in 32 (41%) patients in the public group, and 22 (24%) in the private group (*P =* .01]). Previous treatment with methotrexate was tried in 52 (67%) patients in the public group, and 43 (46%) in the private group (*P =* .007). In the public group, 33 (42%) patients benefited from a compassionate care program from the industry compared with 13 (14%) in the private group (*P <* .0001).

**Table 1.** Baseline Characteristics of the Study Population

**Characteristic (N = 171 except if n - missing)**	**Public^a^ (n = 78)**	**Private (n = 93)**
Age at prescription of advanced therapy, y		
Range	21-91	12-78 ^b^
Mean ± SD (median), y	55 ± 17 (60)	45 ± 13 (46)
Sex, n (%)		
Female	36 (46)	45 (48)
Deprivation Index, material component^c^, n (%) (n - 11)		
≤2	22 (32)	45 (49)
≥3	47 (68)	46 (51)
Deprivation Index, social component^c^, n (%) (n - 11)		
≤2	19 (28)	44 (48)
≥3	50 (72)	47 (52)
Psoriasis subtype, n (%)		
Plaque	64 (82)	84 (90)
Palmoplantar	10 (13)	4 (4)
Pustular	1 (1)	2 (2)
Inverse	1 (1)	0 (0)
Guttate	2 (3)	3 (3)
Psoriasis on difficult-to-treat areas,^d^ n (%)		
Presence	63 (81)	83 (89)
Absence	15 (19)	10 (11)
Comorbidities^e^*,* n (%)		
<2	46 (59)	71 (76)
≥2	32 (41)	22 (24)
Smoking status, n (%)		
Never	53 (68)	70 (75)
Current	13 (17)	14 (15)
Former	12 (15)	3 (10)
Previous conventional therapy^f^, n (%)		
<2	29 (37)	43 (46)
≥2	49 (63)	50 (54)
Rank of prescribed advanced therapy, n (%)		
<2	53 (68)	69 (74)
≥2	25 (32)	24 (26)
Compassion^g^, n (%)	33 (42)	13 (14)
PASI (n - 43)		
Range	4-34	1-26
Mean ± SD (median)	12 ± 7 (10)	11 ± 6 (12)
DLQI (n-1)		
Range	9-30	1-29
Mean ± SD (median)	21 ± 5 (21)	19 ± 5 (20)

Abbreviations: DLQI, Dermatology Life Quality Index; PASI, Psoriasis Area and Severity Index.

^a^ One patient with public insurance for first nations in Canada was included in public group.

^b^ One patient was aged 12 years; all others were >18 years old at index date.

^c^ Material and social deprivation index components are subdivided into quintiles, with socioeconomically privileged individuals within the 1st quintile, to socioeconomically deprived individuals within the 5th quintile.

^d^ Psoriasis on the face, palmoplantar, or genital area.

^e^ Number of comorbidities among diabetes, hypertension, chronic kidney, psoriatic arthritis, dyslipidemia, disease previous lymphoma, and inflammatory bowel disease.

^f^ Including phototherapy, systemic cyclosporine, methotrexate, and acitretin.

^g^ Compassionate care programs from the industry provide a prescribed medication free of charge before its approval by a drug insurance.

A majority of patients were naive to advanced therapy (122 [71%]). During the study period, advanced therapy–naive patients were most frequently prescribed apremilast (29 [24%]), followed by guselkumab (27 [22%]). Individuals naive to advanced therapy had a mean (SD) PASI pre-treatment of 12 (2), compared with 10 (6) in advanced therapy–experimented patients (*P* = .05).

The 2011 Deprivation Index Assignment Program could not assign data to 11 entries (1 unpaired event; 10 indexes not available). The risk of bias was low. There were 22 (32%) patients from the public group and 45 (49%) from the private group who were more socioeconomically privileged (1st, 2nd quintiles of the deprivation index [material component]) (*P =* .03). Similarly, the 1st and 2nd quintiles of the deprivation index [social component] included 19 (28%) patients from the public group, and 44 (48%) from the private group (*P =* .01).

PASI at baseline was not available for 20 (26%) patients in the public group and 23 (25%) patients in the private group. Descriptive and univariate analyses were conducted to characterize this subset of the population; all patients with a missing PASI score had psoriasis on difficult-to-treat-areas (**Supplemental Table S1**).

**Psoriasis severity scores pre-treatment**: In univariate analyses, there was no significant difference between the groups in PASI, DLQI, and IGA pre-treatment. Results from descriptive and univariate analyses are summarized in **Table 2**.

**Table 2.** Results from Univariate Analyses on Psoriasis Severity Scores and Time to Start of Advanced Therapy^a^

**Outcome Variable**	**Public (n=78)**	**Private (n=93)**	**Association Measure (95% CI)**	***P* Value**
Pre-treatment psoriasis severity scores				
PASI (n - 43)	12 ± 7 (10), 4-34	11 ± 6 (12), 1-26	MD 0.41 (-1.80-2.61)	.72
DLQI (n - 1)	21 ± 5 (21), 9-30	19 ± 5 (20), 1-29	MD 1.07 (-0.46-2.60)	.17
IGA	3 ± 1 (3), 2-4	3 ± 1 (3), 1-4	MD -0.07 (-0.30-0.15)	.53
Post-treatment psoriasis severity scores				
PASI (n - 57)	3 ± 4 (2), 0-18	3 ± 4 (1), 0-14	MD 0.08 (-1.36-1.52)	.91
DLQI (n - 26)	6 ± 7 (2), 0-27	6 ± 8 (1), 0-28	MD -0.02 (-2.47-2.44)	.99
IGA (n - 1)	1 ± 1 (1), 0-4	1 ± 1 (1), 0-4	MD 0.01 (-0.32-0.34)	.93
Treatment response (n - 69)				
|PASI ∆|	6 ± 7 (2)	6 ± 7 (2)	MD 0.69 (-1.74-3.11)	.58
PASI50	39 (87)	42 (74)	RR 1.18 (0.97-1.43)	.11
PASI75	26 (58)	35 (61)	RR 0.94 (0.68-1.30)	.71
PASI90	17 (38)	25 (44)	RR 0.86 (0.54-1.39)	.54
Time from prescription to start of advanced therapy, days (n - 6)				
Log(delay)^b^	3.20 ± 0.79 (3.24), 0.69-5.17	3.12 ± 0.78 (3.18), 1.10-4.94	MD -0.08 (-0.32-0.16)	.51
Delay	32 ± 26 (26), 2-175	24 ± 25 (24), 3-140	—	—

Abbreviations: DLQI, Dermatology Life Quality Index; IGA, Investigator’s Global Assessment; MD, mean difference; PASI, Psoriasis Area and Severity Index; RR, relative risk.

^a^ Continuous variables presented as mean ± SD (median), range; dichotomic variables as n (%).

^b^ The variable delay to first dose of advanced therapy was log-transformed to control for abnormality of the distribution and extreme variables. The *t*-test statistics were performed on log-transformed data.

In multivariate analyses, we observed a higher score of 2.79 in PASI pre-treatment for patients from the public group compared with private (*P =* .03), when the covariates age at prescription, deprivation index (social component), and compassion care remained constant (**Table 3**). Our confidence in these results is low due to the poor overall fit of the model (*P* value from *F* test = .058). The coefficient of determination is low; the model explained 4.35% of variability on PASI pre-treatment. Even if the *P* value from the overall *F* test was significant, we considered a difference of 2.79 in PASI pre-treatment to be of low clinical relevance. Moreover, our study was not designed to detect a clinical difference less than 5 in PASI pre-treatment. All assumptions for linear regression models were verified and fulfilled.

**Table 3.** Multiple Linear Regression Adjusted for Confounding Covariables

**Independent Variable**	β	**95% CI**		***P* Value**
		**Upper Limit**	**Lower Limit**	
PASI pretreatment (*P* value from overall *F* test, .0580; adj *R*,^2^ 4.35%)				
Type of drug insurance plan	2.79	0.22	5.46	0.034
Age (y) at prescription	-0.07	-0.15	0.002	0.057
Deprivation Index, social component^a^	2.41	0.08	4.74	0.043
Compassion^b^	-1.45	-4.23	1.33	0.305
DLQI pretreatment (*P* value from *F* test, <.0001; adj *R^2^,* 20.21%)				
Type of drug insurance plan	1.35	-0.19	2.88	0.085
Age at prescription	-0.02	-0.07	0.02	0.325
Deprivation Index, social component^a^	-0.49	-1.97	0.99	0.513
Advanced therapy^c^	-0.26	-0.63	0.12	0.183
Previous advanced therapy-exposure^d^	-4.53	-6.21	-2.85	<.0001
Treatment response |∆ PASI| (*P* value from *F* test, .0221; adj *R^2^,* 8.48%)				
Type of drug insurance plan	3.69	0.91	6.47	0.010
Age at prescription	-0.11	-0.19	-0.03	0.009
Deprivation Index, social component^a^	1.86	-0.60	4.32	0.137
Deprivation Index, material component^a^	1.19	-1.36	3.74	0.357
Time to renewal	0.52	-0.06	1.10	0.079
Time between prescription and biologic initiation^e^ (*P* from *F* test, .35; adj *R*,^2^ 0.78%)				
Type of drug insurance plan	0.06	-0.25	0.37	0.699
Age at prescription	0.01	-.004	0.01	0.259
Deprivation Index, social component^a^	-0.02	-0.30	0.26	0.889
Deprivation Index, material component^a^	0.08	-0.19	0.36	0.541
Psoriasis subtype	-0.04	-0.23	0.14	0.628
Psoriasis on difficult-to-treat areas^f^	0.37	-0.02	0.76	0.066
Previous conventional treatments^g^	-0.12	-0.39	0.14	0.349
Previous exposure to advanced therapies	-0.16	-0.45	0.13	0.267
Compassion care^b^	-0.04	-0.35	0.28	0.820
Time to renewal	-0.03	-0.09	0.03	0.347

Abbreviations: CI, confidence interval; DLQI, Dermatology Life Quality Index; PASI, Psoriasis Area and Severity Index.

^a^ Categorized quintile of the regional Canadian Deprivation Index. Absence of multicollinearity was verified and fulfilled on full models.

^b^ Compassionate care programs from the industry provide a prescribed medication free of charge before its approval by a drug insurance.

^c^ Prescribed advanced therapy (discrete data).

^d^ Previous exposure to advanced therapy (dichotomous data).

^e^ The variable delay was log-transformed. Results from *t*-test on log[delay] are presented.

^f^ Psoriasis on the face, palmoplantar, or genital area.

^g^ Including phototherapy, systemic cyclosporine, methotrexate, and acitretin.

In multivariate analyses, no statistically or clinically significant difference in DLQI pre-treatment was found between public vs private groups (*P =* .085) (**Table 3**). At least 1 independent variable of adjustment was associated with DLQI pre-treatment (overall *F* statistic, 9.00; *P <* .0001). The model explained 20% of variability on DLQI pre-treatment (adjusted R^2^ 0.2021). The association was driven by previous exposure to advanced therapies; if all independent variables remain constant, DLQI pre-treatment is expected to decrease by 4.53 in patients advanced therapy-experimented compared with patients naive to advanced therapy (*P <* .0001).

**Psoriasis severity at renewal**: In univariate analyses (**Table 2**), there was no significant difference between public vs private groups in PASI, DLQI, and IGA scores at time of advanced therapy renewal (*P>*.05).

**Treatment response**: In univariate analyses (**Table 2**), there was no significant difference between public vs private groups in treatment response (*P>*.05). In multivariate analyses, treatment response was higher by 3.69 in publicly insured patients compared with private (*P =* .01), if the covariables age at prescription, deprivation index (social and material components), and time to renewal of prescription remain constant (**Table 3**). The association was driven by the predictor variable of interest type of drug insurance, and the covariate age at prescription. We are confident that at least 1 estimate parameter was associated with treatment response (*F* statistic, 2.78; *P =* .02). However, the coefficient of determination was low; the model could explain 8.48% of variability on treatment response.

**Achievement of PASI50, PASI75, and PASI90**: In univariate analyses (**Table 2**), there was no significant difference between the public vs private groups in achievement of PASI50, PASI75, and PASI90 (*P>*.05).

**Delay from index date to first dose of advanced therapy**: In univariate and multivariate analyses, there was no significant difference between the groups in log[delay] (**Table 2**, **Table 3**). None of the model variables were associated with log[delay] (overall *F* statistic, 1.12; *P =* .35). The coefficient of determination was very low; the model explained only 0.78% of variability on log[delay].

## DISCUSSION

We observed a high prevalence of psoriasis on difficult-to-treat areas, namely the face, genitalia, and palmoplantar areas (85.4%). In comparison, previous studies reported up to 46% genital involvement^23^ and a prevalence of facial psoriasis in 67.8% of patients.^24^ This discrepancy could be due to the nature of our variable, which captured multiple areas of psoriasis on the body surface. When recognizing the need for effective systemic therapy for moderate-to-severe psoriasis in a resource-limited setting, clinicians might also favor the use of a dichotomic, less time-consuming criterion.

Compassionate care for advanced therapy in psoriasis refers to prescribed medication, provided free of charge, usually while awaiting approval from an insurer. We observed 33 (42%) patients in the public group who benefited from compassionate care programs from the industry, compared with 13 (14%) patients in the private group (relative risk [RR] 3.03; 95% confidence interval [CI], 1.72–5.34; *P <* .0001).

Prescription drug insurance is a complex subject, and nuances must be made regarding long-term compassion (or free goods), and short-term compassionate programs by the industry (or bridging). Considering the high prevalence of short-term compassion in our study population (46/171; 26.9%), our results highlight the support provided by the industry to initiate biologic therapy in psoriasis patients. This temporary financial support appears complementary to prescription drug insurance plans. The high prevalence of compassionate care in publicly funded insurance programs may contribute to mitigate differences between the groups, especially regarding time to initiation of advanced therapy. Importantly, if compassionate care programs were to be abolished, publicly insured patients could experience increased delays before the start of an advanced therapy.

The nature of our public prescription drug insurance plan itself accounts for differences observed in clinical and demographic characteristics. Individuals covered by the public prescription drug insurance plan in Québec are either aged 65 years or older, subscribers of the social assistance program, or unable to benefit from a private drug insurance plan through their workplace. The mean patient age in the public group was 10.13 years older than in the private group (95% CI, 5.52-14.74; *P <* .0001). Individuals in the public group were also less socioeconomically privileged with regard to the material (RR = 0.66; 95% CI: 0.45-0.99; *P =* .03) and social (RR *=* 0.59; 95% CI: 0.39-0.91; *P* = .01) components of the Canadian Deprivation Index.

We observed patients with psoriasis during a mean (SD) period of 5 (2) months from prescription of advanced therapy to renewal of treatment. Globally, a significant improvement in psoriasis severity scores was observed: PASI50, PASI75, and PASI90 were achieved in 79.4%, 59.8%, and 41.2% of the study participants, respectively.

We compared patients in public vs private prescription drug insurance groups and did not observe a difference in PASI pre-treatment (12 [7] vs 11[6]; *P =* .72). In univariate analyses, severity scores observed at index date and at renewal, treatment response, and log[delay] were also not different between groups (*P>* .05) (**Table 2**).

Results from multivariate analyses on the relationship between PASI pre-treatment and type of drug insurance should be interpreted cautiously. The overall fit of the model is not statistically significant based on the *F* test’s *P* value (*P =* .06). Similarly, we must interpret with caution the model studying the adjusted relationship between log[delay] and type of drug insurance since it has an overall *F*-test *P* value that is not statistically significant (*P =* .35) and it does not include statistically significant individual *t-*test statistics (**Table 3**).

Limitations of this study are mainly related to its retrospective design.^25^ Our convenience sample from a tertiary care academic hospital in Québec City raises the possibility of selection bias, favoring more severe cases of psoriasis. An information bias arises from the variable PASI pre-treatment, which was missing in 43/171(25%) patients, likely due to the use of psoriasis on difficult-to-treat areas as an alternative reimbursement criterion. The remaining population size was sufficient to detect a difference of 5 points in PASI scores between groups, using 80% power and a 5% level of significance. The inclusion of patients with previous exposure to advanced therapies could underestimate severity scores and treatment response. Unlike previous work using type of drug insurance as an independent variable, study strengths include adjustments for many important covariates, including socioeconomic status, compassion, comorbidities, psoriasis on difficult-to-treat areas, and previous exposure to biologic, which contributed to limit confusion bias. The use of validated scores to measure psoriasis severity is another strength of this study.

## CONCLUSIONS

This retrospective cohort study was conducted in 171 patients with psoriasis treated by advanced therapy in a Canadian tertiary academic care center. We compared psoriasis degree of severity pre-treatment and at renewal of advanced therapy, treatment response, and delay to first dose between the public and private groups. Importantly, prescribers of advanced therapies for psoriasis can be reassured, as we found no statistically and clinically significant difference in access to advanced therapies between publicly and privately insured patients. Regarding the access to advanced therapies in patients with psoriasis, our study highlights a precarious safety net, particularly in publicly insured patients. A high prevalence of short-term compassionate care programs is probably beneficial for patients, as they are provided with rapid and effective treatment for their chronic, distressful disease. However, compassionate care programs might camouflage a deeper problem in access to prescription drugs in Canada and worldwide, pointing toward the increasing economic burden from the high cost of advanced therapies and questioning the sustainability of their coverage. To clarify the effect of compassionate care programs on patients with public and private drug insurance plans, further studies should plan statistical inferences from multivariate analyses.

The frequent utilization of psoriasis on difficult-to-treat areas as an alternative reimbursement criterion, on the other hand, raises a concern regarding choice of the appropriate regulations to access advanced therapies. The authors favor a more flexible criterion that would rely on clinicians’ clinical judgment of biopsychosocial impacts of psoriasis.

## Supplementary Material

Online Supplementary Material
