# Supplementary material for: Drug Insurance and Psoriasis Severity: A Retrospective Cohort Study
Source: J Health Econ Outcomes Res. 2025 Feb 7;12(1):51–7. doi: 10.36469/001c.127820 (PMC11807372; doi:10.36469/001c.127820)
Supplement: Online Supplementary Material [file jheor_2025_12_1_127820_265171.pdf]

Journal of Health Economics and Outcomes Research

Online Supplementary Material

Drug Insurance and Psoriasis Severity: A Retrospective Cohort Study. *JHEOR*. 2025;12(1):51-57. doi:10.36469/jheor.2025.127820

Table S1: Exploratory Analysis of Patient Characteristics (n=171 Except If [n - Missing])

This supplementary material has been provided by the authors to give readers additional information about their work.

This is an open-access article distributed under the terms of the Creative Commons Attribution 4.0 International License (CCBY-4.0). View this license's legal deed at http://creativecommons.org/licenses/by/4.0 and legal code at http://creativecommons.org/licenses/by/4.0/legalcode for more information.

Table S1. Exploratory Analysis of Patient Characteristics (n=171 Except If [n - Missing])

**Characteristic (n = 171 except if n-missing)**	**PASI Available (n = 128)**	**PASI Missing (n = 43)**	***P* Value**
Psoriasis on difficult-to-treat areas, n (%)			
Presence	103 (80)	43 (100)	.002
Absence	25 (20)	0 (0)	
Exposure to advanced therapy, n (%)			
Advanced therapy exposure	43 (34)	6 (14)	.014
Advanced therapy–naive	85 (66)	37 (86)	
Log(delay) (n - 6)[a]			
Range	0.69–5.17	2.20–4.84	.009
Mean ± SD (median)	3.07 ± 0.81 (3.05)	3.44 ± 0.65 (3.38)	
**Summary of Patients With Compassion and Difficult-to Treat Areas**			
	**Compassion (n = 44)**	**No Compassion (n = 121)**	***P* Value**
Log(delay)[b] (n - 6)			
Range	0.69–5.16	1.10–4.94	.469
Mean ± SD (median)	3.08 ± 0.85 (3.13)	3.18 ± 0.76 (3.26)	
	**Psoriasis on Difficult-to-Treat Areas (n = 140)**	**No Psoriasis on Difficult-to-Treat Areas (n = 25)**	***P* Value**
Log(delay)[b] (n - 6)			
Range	0.69–5.16	1.10–3.89	.056
Mean ± SD (median)	3.20 ± 0.78 (3.24)	2.88 ± 0.77 (3.00)	
	**Advanced Therapy–Naive (n = 117)**	**Advanced Therapy Exposure (n = 48)**	
Log(delay) (n - 6)			
Range	0.69–5.16	1.61–4.94	.093
Mean ± SD (median)	3.22 ± 0.80 (3.30)	3.00 ± 0.71 (3.02)	

Abbreviations: DLQI, Dermatology Life Quality Index.

[a] Untransformed mean (SD) values in days for delay: 38 (24) days PASI available, vs 30 (26) days in PASI-missing patients. Student's *t* tests were conducted on log(delay) and *P* values were robust.

[b] Untransformed mean (SD) values in days for delay: 31 (31) in patient who benefited from compassion, vs 31 (24) without compassion; 33 (27) in patients with psoriasis on difficult-to-treat area, vs 23 (14) days without psoriasis on difficult-to-treat areas.
